# Supplementary material for: Systematic identification of intron retention associated variants from massive publicly available transcriptome sequencing data
Source: Nat Commun. 2022 Sep 29;13:5357. doi: 10.1038/s41467-022-32887-9 (PMC9522810; doi:10.1038/s41467-022-32887-9)
Supplement: Supplementary file 1 — Supplementary Information [file 41467_2022_32887_MOESM1_ESM.pdf]

Supplementary Information

Systematic identification of intron retention associated variants from massive publicly available transcriptome sequencing data

Yuichi Shiraishi, Ai Okada, Kenichi Chiba, Asuka Kawachi, Ikuko Omori, Raúl Nicolás Mateos, Naoko Iida, Hirofumi Yamauchi, Kenjiro Kosaki, Akihide Yoshimi

a

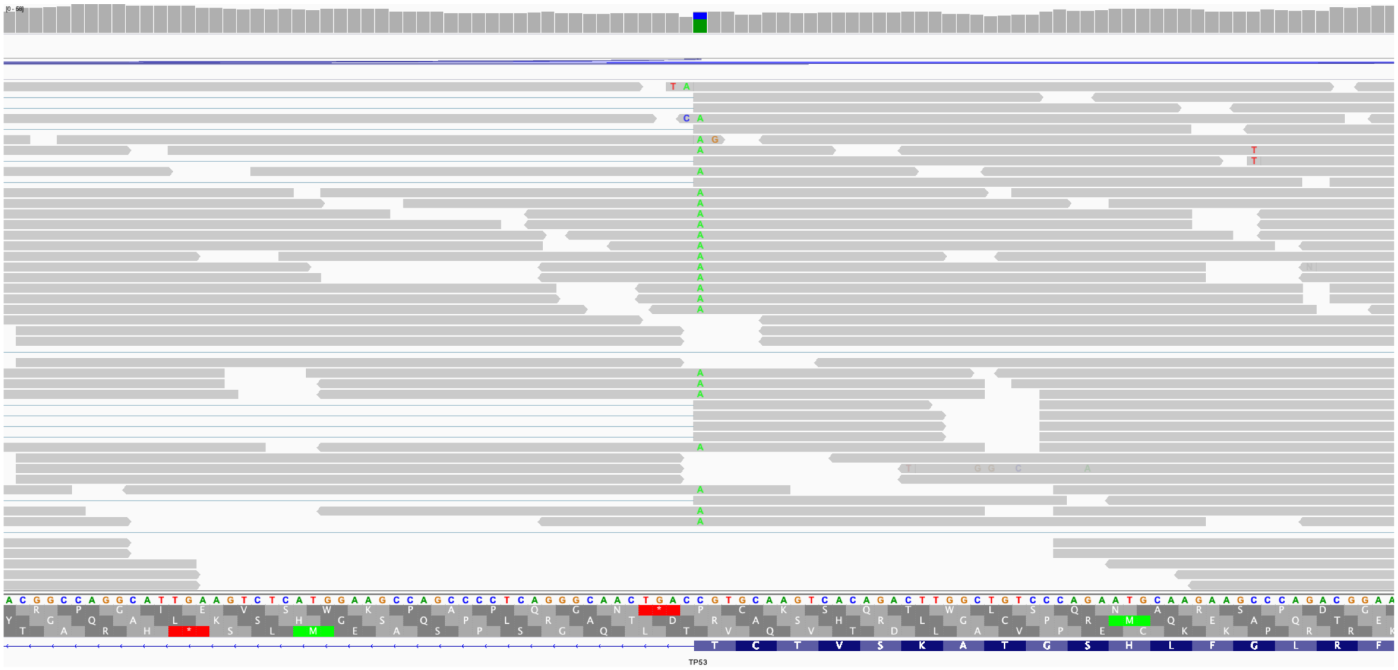

b

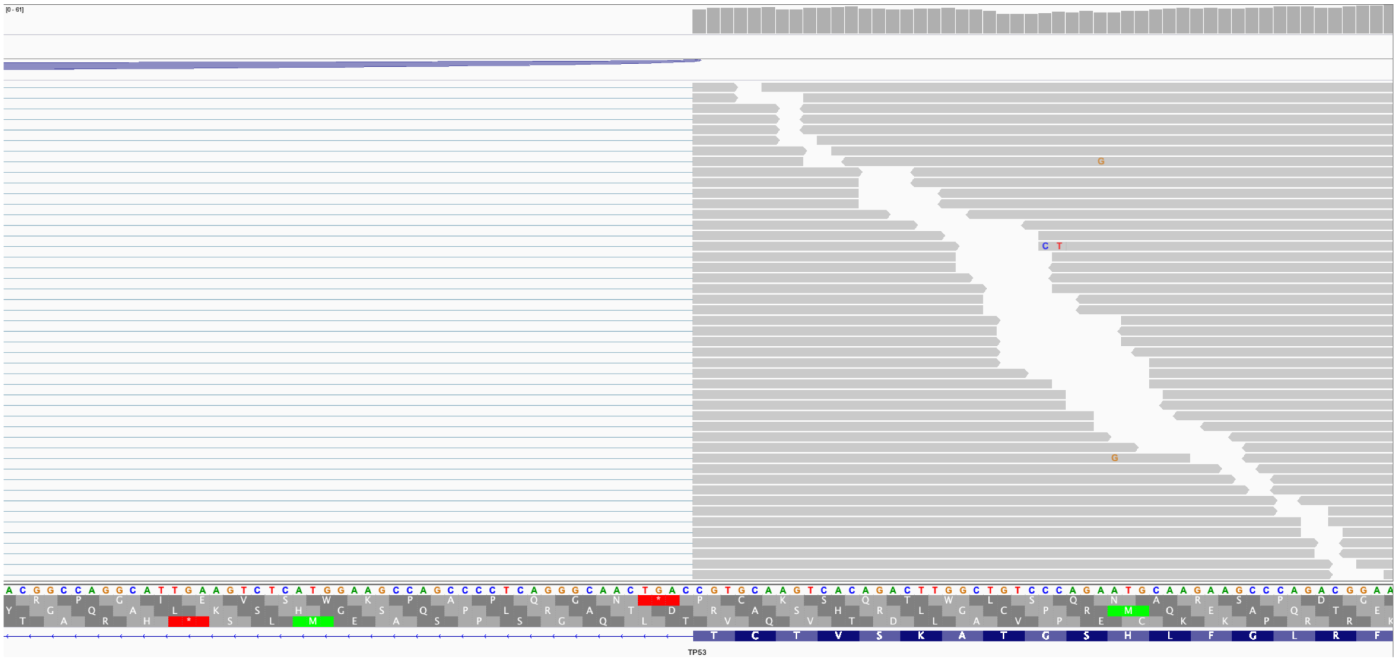

Supplementary Figure 1: **Example of alignment status around intron retention associated variants by the depiction of Integrative Genomics Viewer.** Here, the alignment views of the *TP53* exon 4 donor site were shown for (a) the sample having the corresponding IRAVs (chr17:7675994 C>A) as well as (b) one with no IRAVs at that splice site.

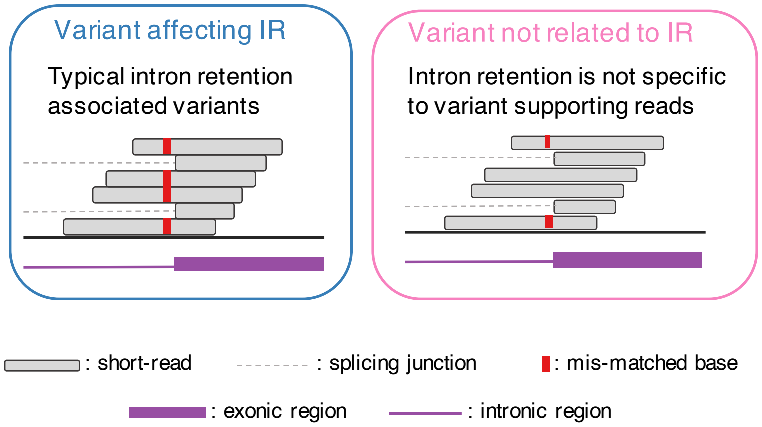

Supplementary Figure 2: **A conceptual diagram of how alignment and mismatch bases can be shaped at the exon-intron boundary by IRAVs.** In the left panel (which is an example of typical IRAVs), there are four intron retention reads, and all the intron retention reads support the mismatch base of the target variant. We keep these variants as IRAVs. On the other hand, in the right panel, there are four intron retention reads, and two of them did not support the mismatch base. In those cases, these candidates are removed. See the subsection of “Detection of intron retention associated variants from CRAM format files” in the Method section.

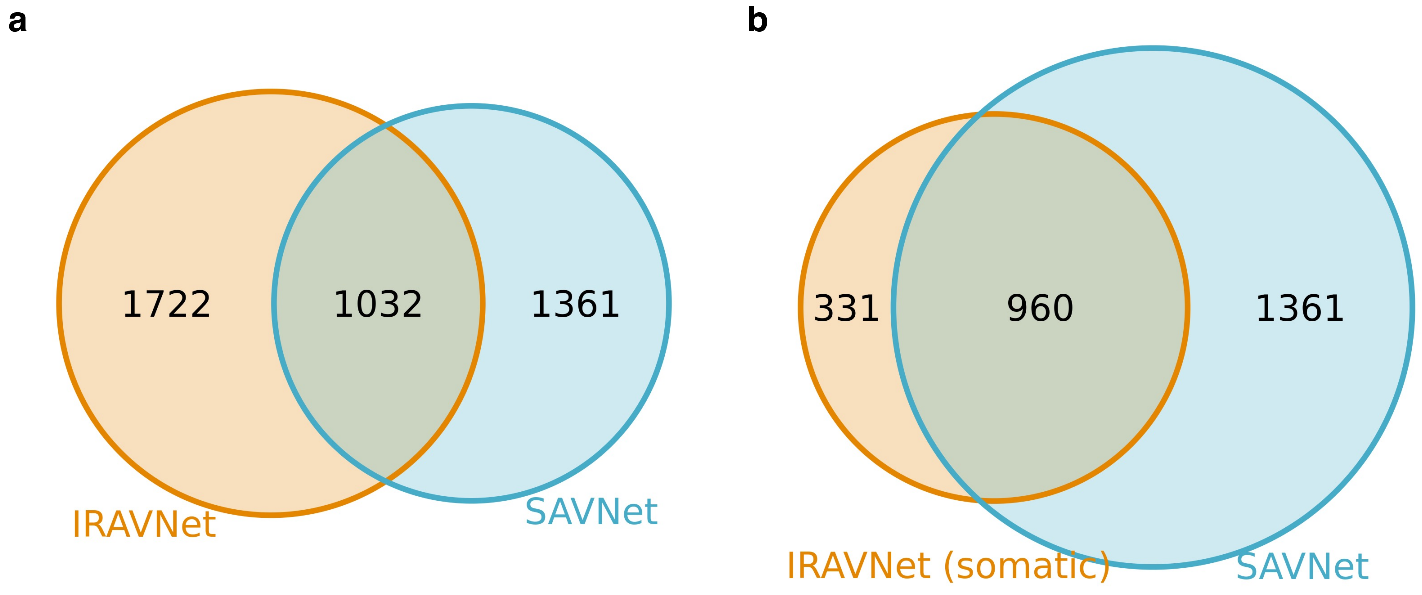

Supplementary Figure 3: **Venn diagrams showing the overlap of intron retention associated variants identified by the current (IRAVNet) and previous approach (SAVNet).** (a) The overlaps with all the IRAVs detected by IRAVNet are depicted. (b) The overlap with IRAVs restricted to be those classified into “somatic” via tumor and matched control exome sequencing data are shown, since IRAVNet also detects germline variants whereas SAVNet only focuses on somatic variants. Splicing associated variants identified by SAVNet were restricted to those causing intron retention.

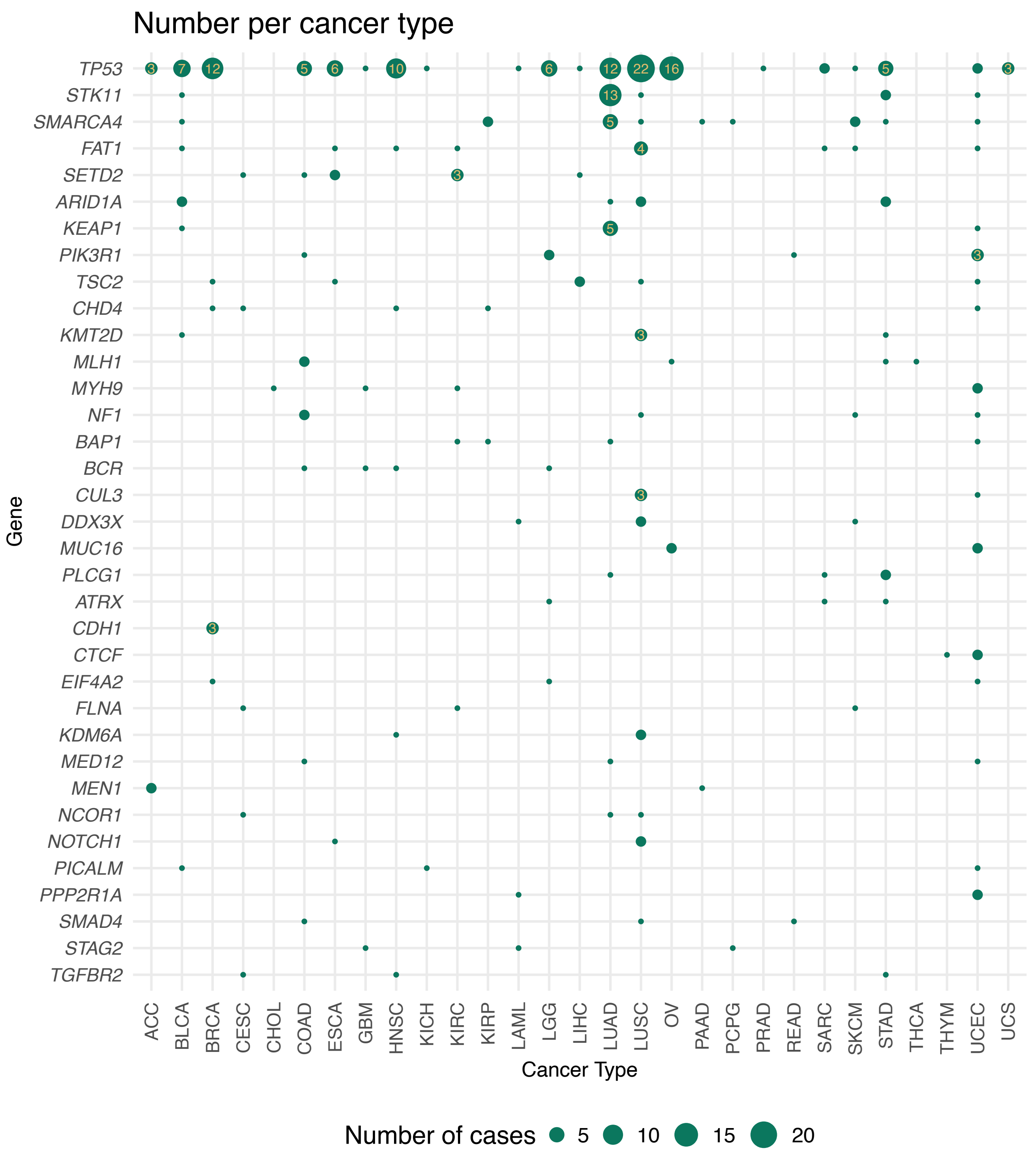

Supplementary Figure 4: **Landscape of IRAVs in frequent cancer-related genes (total number ≥ 3) across cancer types.** The font size indicates the number of affected samples. Genes are sorted by the total number of IRAVs in all cancer types. See also Figure 2d.

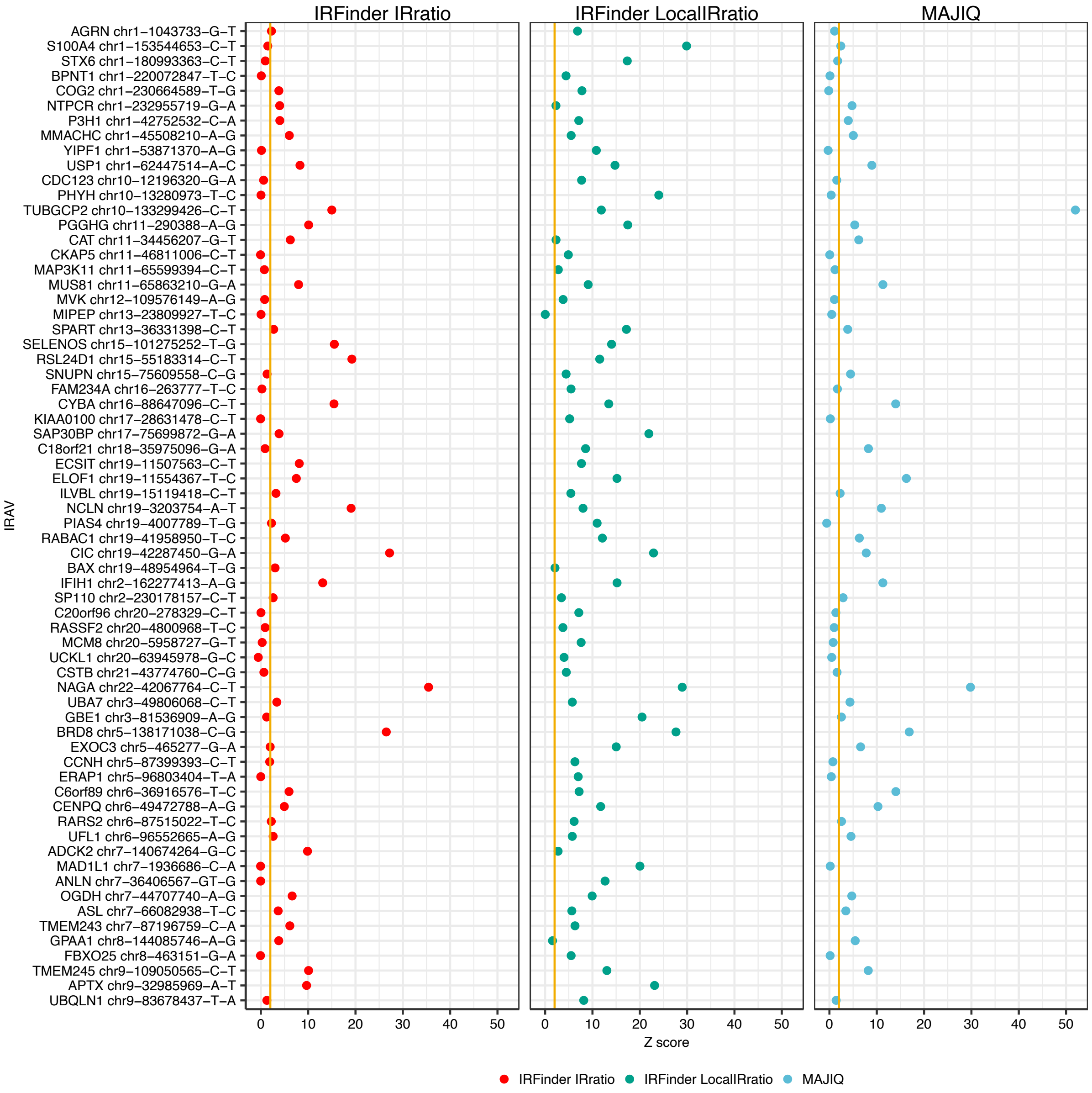

Supplementary Figure 5: Effect of intron retention for the IRAVs identified in GEUVADIS dataset measured by Z-value statistics. For each IRAV, the Z-values (for IRFinder IRratio, IRFinder LocalIRratio, and MAJIQ) comparing the ratios of intron retention between samples with and without the IRAV were computed.

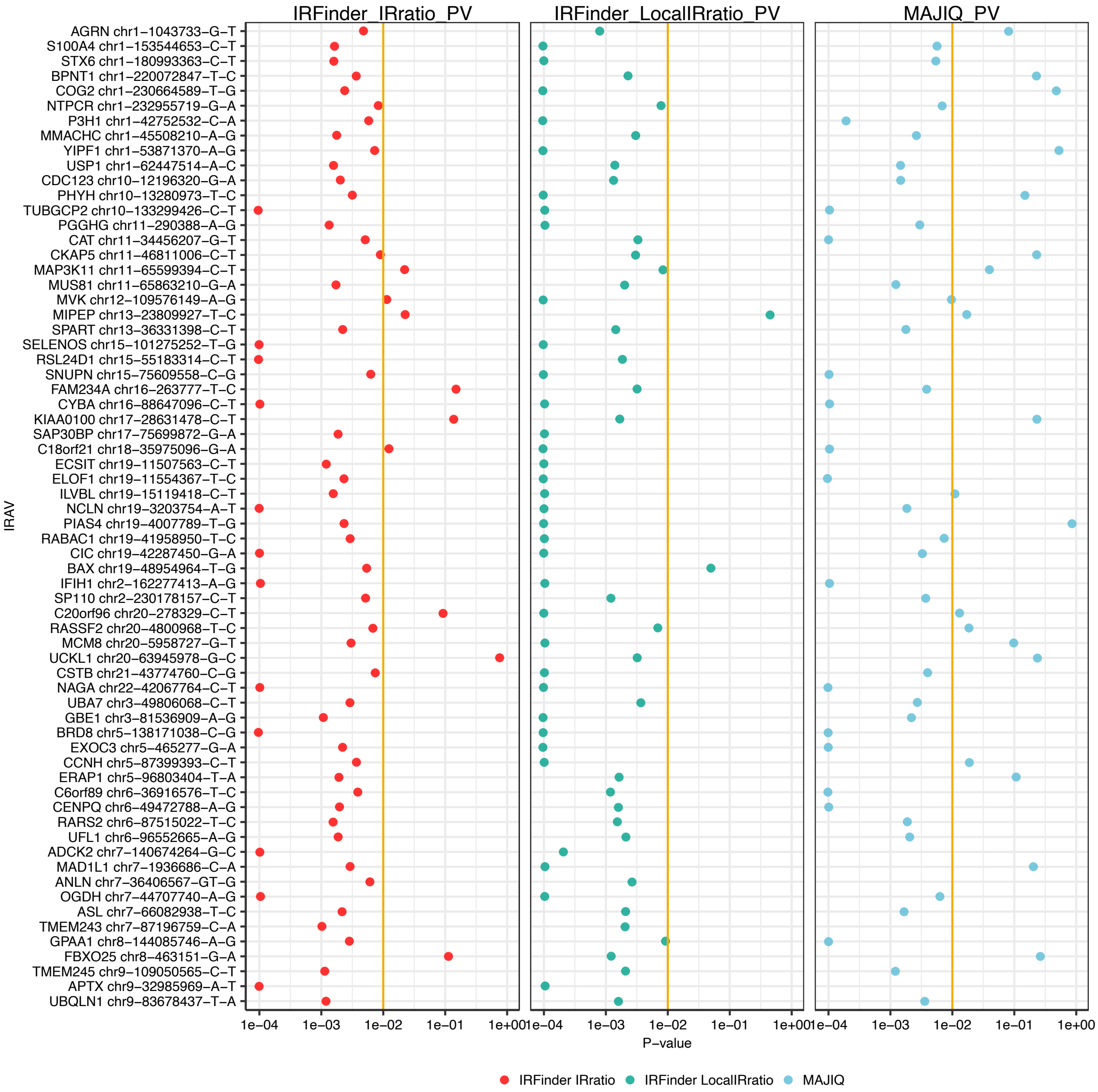

Supplementary Figure 6: Effect of intron retention for the IRAVs identified in GEUVADIS dataset measured by permutation test. For each IRAV, the p-values (for IRFinder IRratio, IRFinder LocalIRratio, and MAJIQ) based on a permutation test (one-sided, without any p-value adjustment) comparing the ratios of intron retention between samples with and without the IRAV were computed.

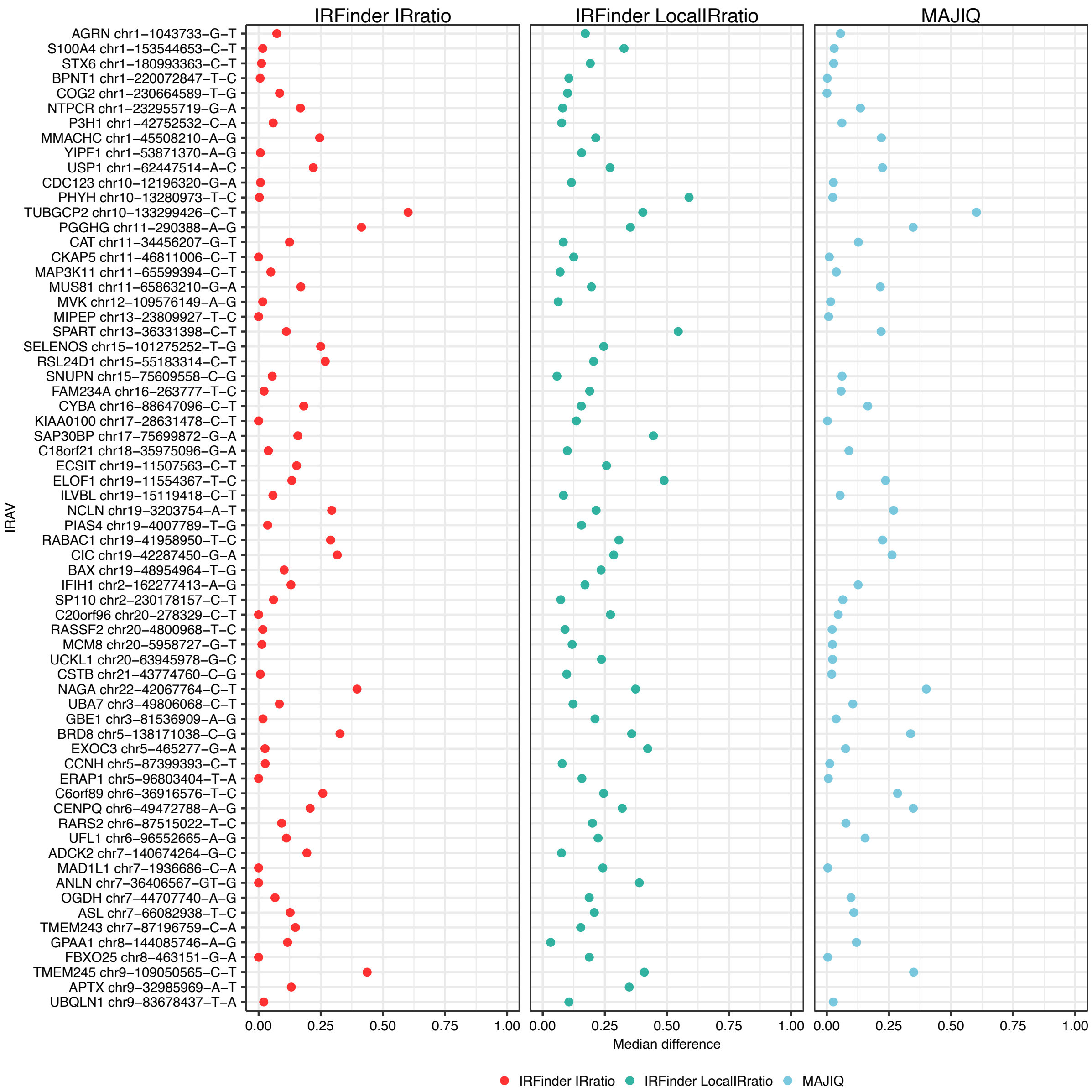

Supplementary Figure 7: Effect of intron retention for the IRAVs identified in GEUVADIS dataset measured by the difference of median values. For each IRAV, the difference of median values (for IRFinder IRratio, IRFinder LocalIRratio, and MAJIQ mean_psi_lsv_junction) between samples with and without the IRAV was computed. No adjustment was made to the output p-values of MAJIQ HET.

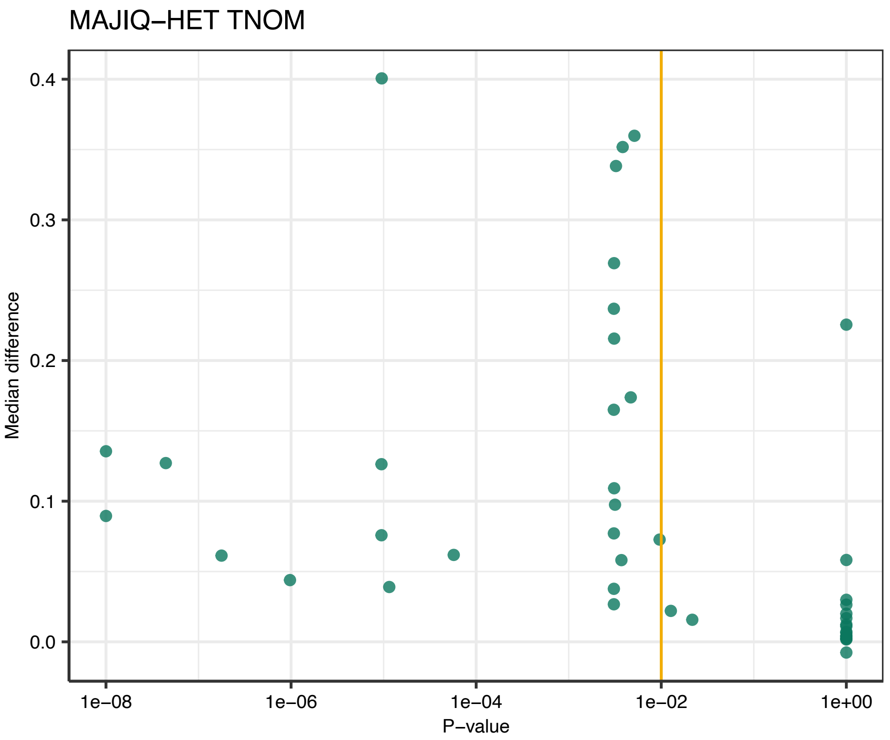

Supplementary Figure 8: Effect of intron retention for the IRAVs identified in GEUVADIS dataset measured by MAJIQ HET. For each IRAV, the significance of the difference in percent spliced in (PSI) was measured via the p-value (x-axis) and median difference (y-axis).

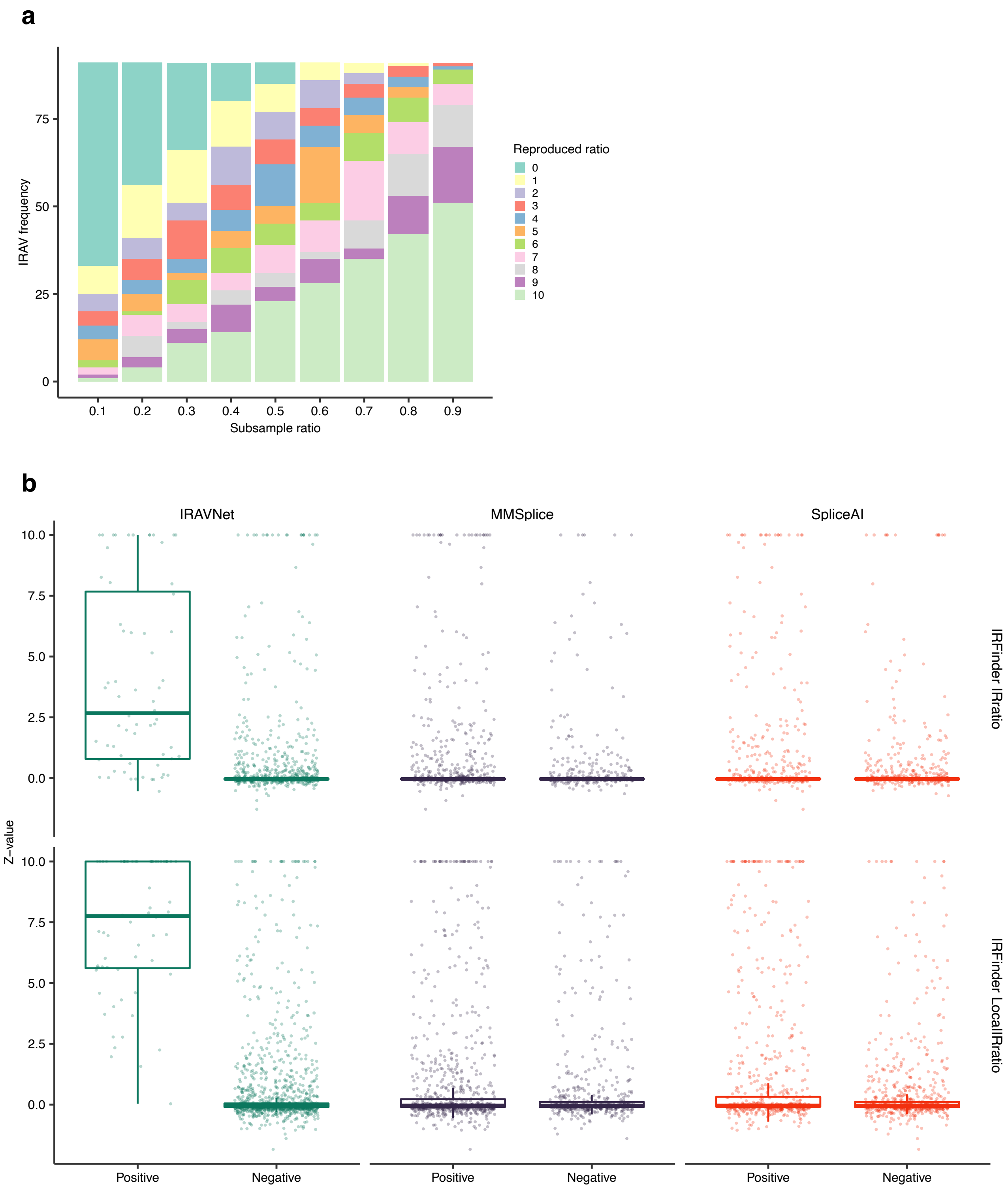

Supplementary Figure 9: Evaluation of IRAVNet robustness and comparison with machine learning-based prediction approaches. (a) The bar plot shows how many times IRAVs were redetected out of 10 attempts with various subsampling ratios. For example, when the subsample ratio is 0.9, about half of the IRAVs were identified in all 10 trials. (b) The Z-values were plotted for the variants that were positive or negative for IRAVNet, MMSplice, and SpliceAI. The ends of the boxes indicate lower and upper quartiles; center line, median; whiskers, maximum and minimum values within 1.5×IQR from the edges of the box, respectively. See Method section for details on the analysis.

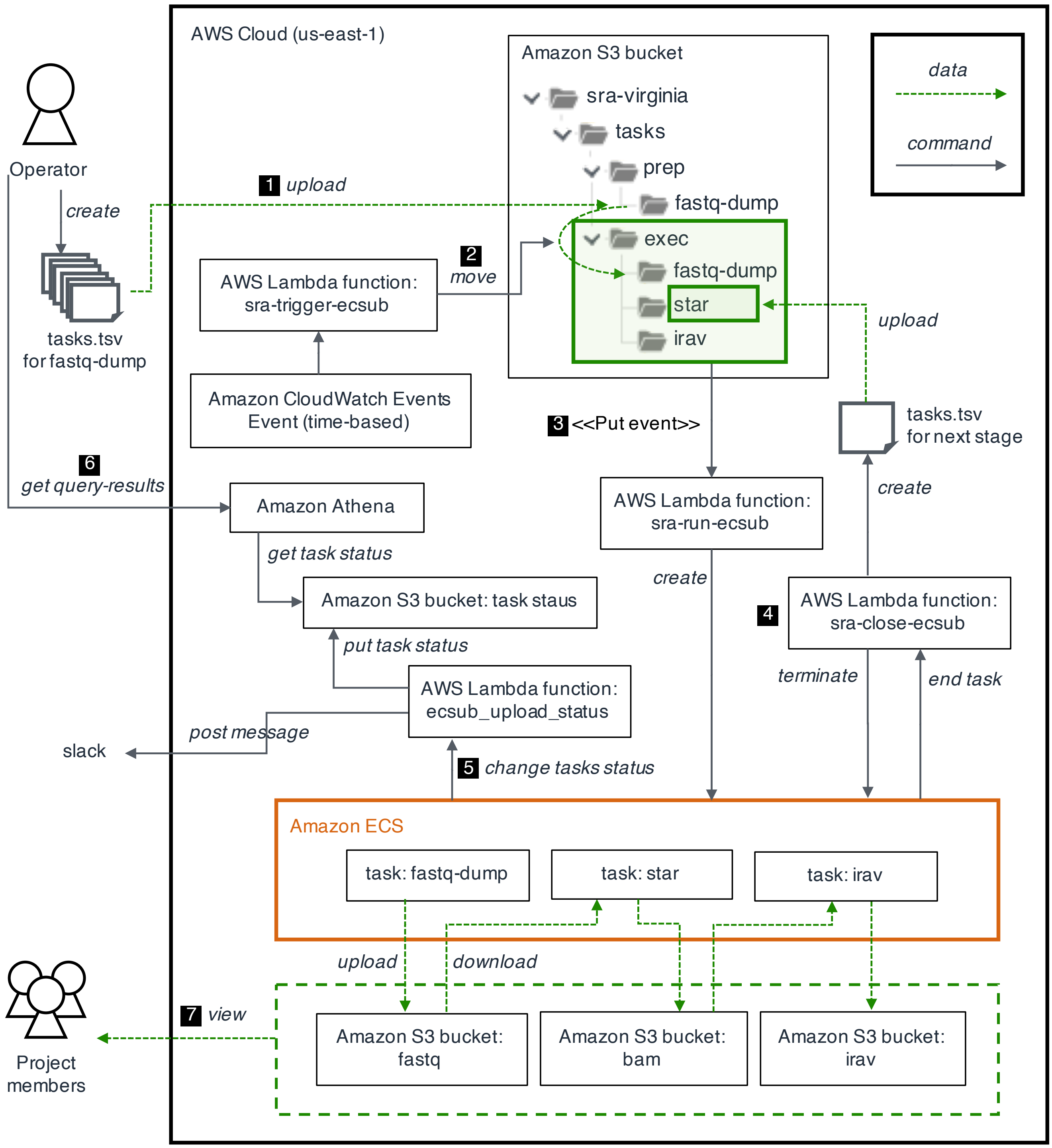

Supplementary Figure 10: A workflow for detecting IRAVs using Amazon Web Service. We performed three tasks for each transcriptome sequencing data; The tasks:fastq-dump performs downloading from Sequence Read Archive and converts the sequence data into FASTQ format. The task:star performs alignment to the reference genome using STAR. The task:irav performs IRAVNet to identify IRAVs. A serverless framework (Amazon Lambda) controls the job execution, which detects the placement meta-data describing the sample to analyze and launch the batch job using ecsub package (a wrapper for Amazon Elastic Container Service).

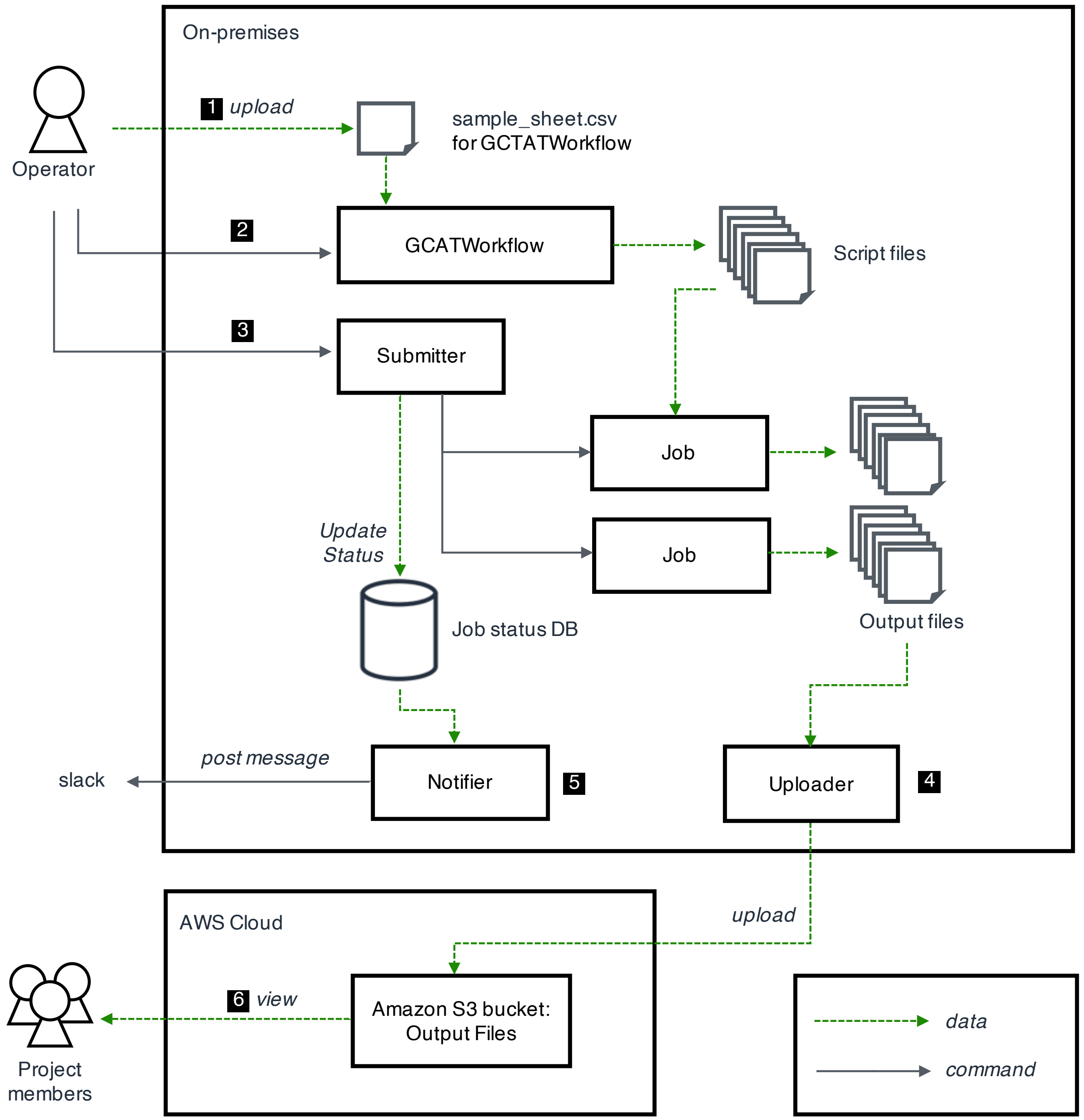

Supplementary Figure 11: A workflow for detecting IRAVs using Univa Grid Engine. We developed snakemake based pipeline, GCATWorkflow, to execute downloading FASTQ file, alignment to the reference genome, and IRAV detection in order. Also, two background jobs (uploader and notifier) upload the output to Amazon S3 and notify the success or failure to Slack, respectively.

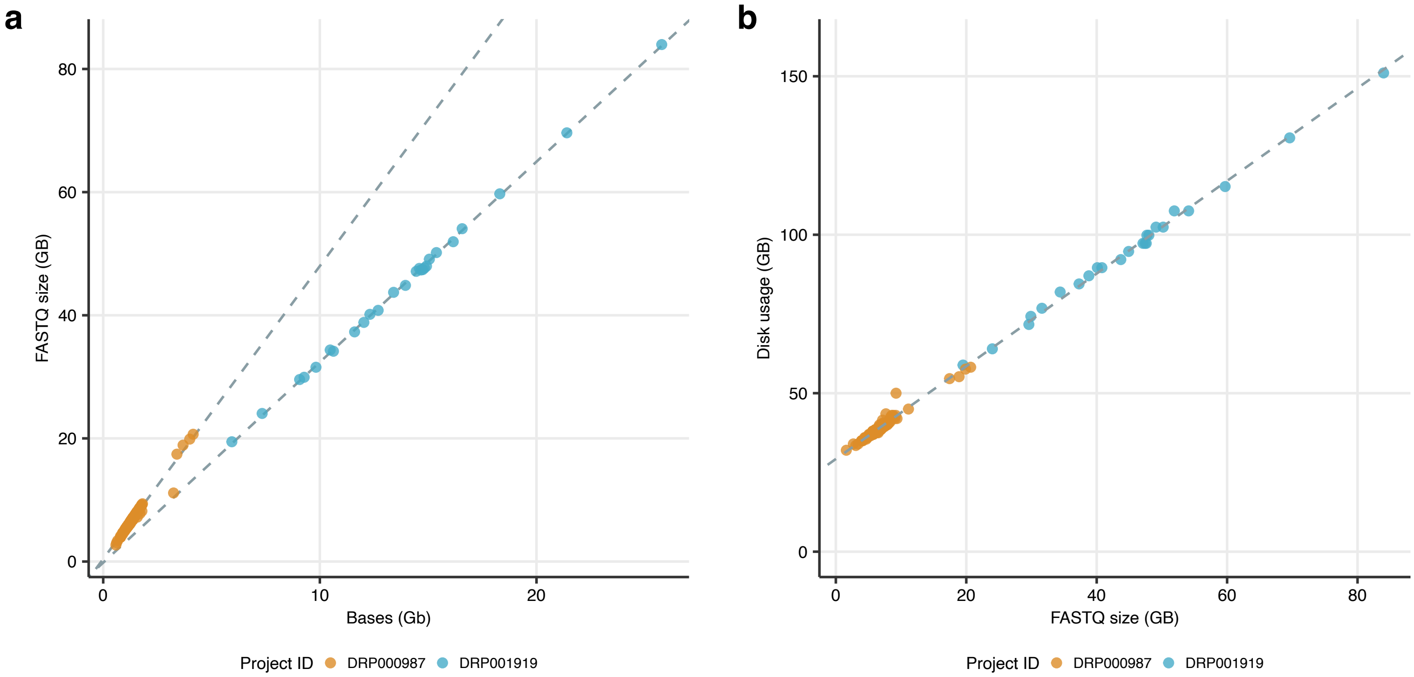

Supplementary Figure 12: Evaluation of data size and computing resource. (a) The relationships between the number of bases extracted from meta-data file from Sequence Read Archive and actual FASTQ file size. The points that represent each sample line up almost exactly with the regression line for each project. (b) Relationships between FASTQ file size and disk usage when performing STAR alignment. The read lengths were 75bp and 100bp for DRP000987 and DRP001919, respectively.

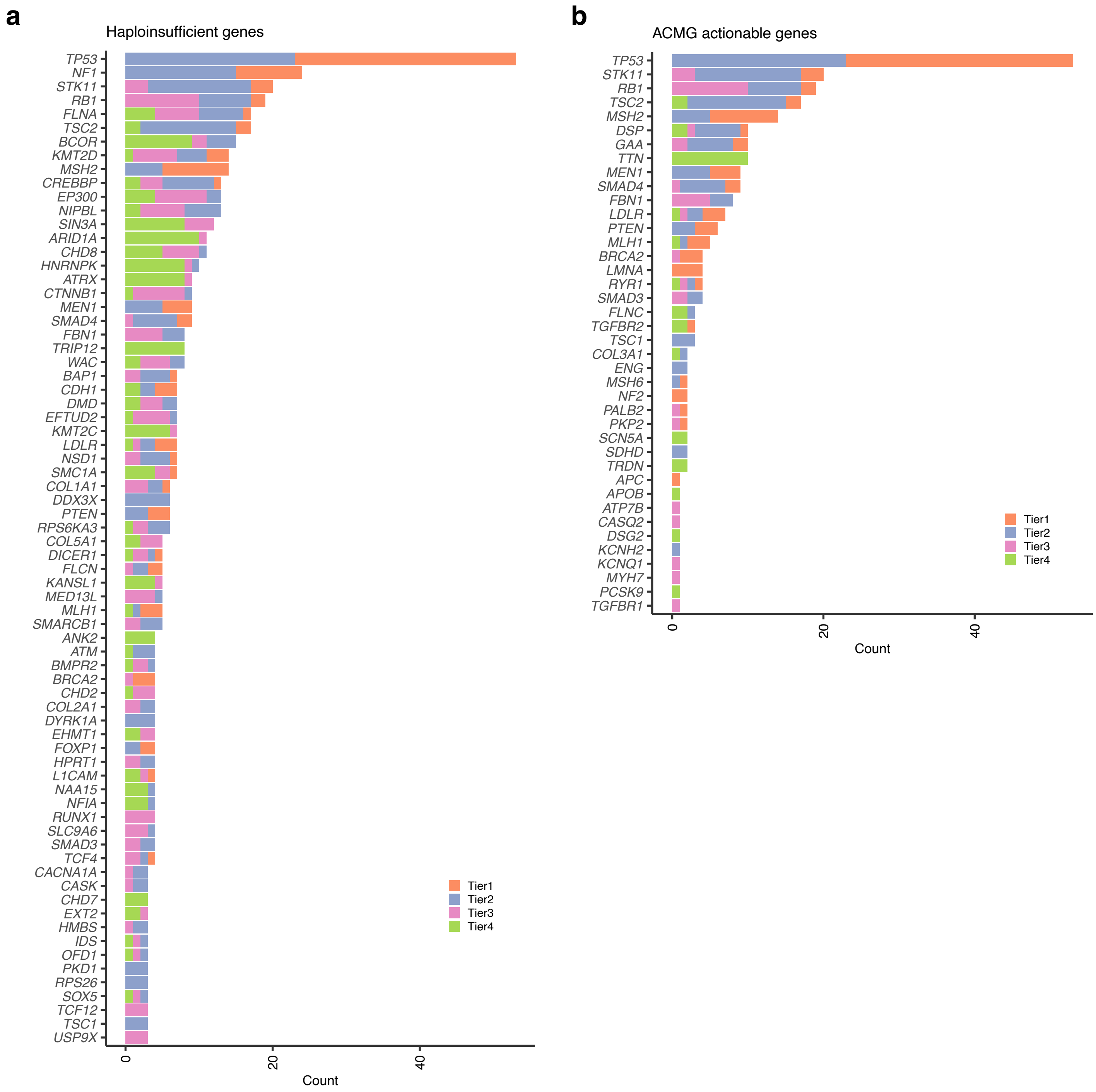

Supplementary Figure 13: The landscape of putative pathogenic IRAVs in (a) haploinsufficient genes and (b) ACMG actionable genes. Genes are sorted by the total number of distinct variants,

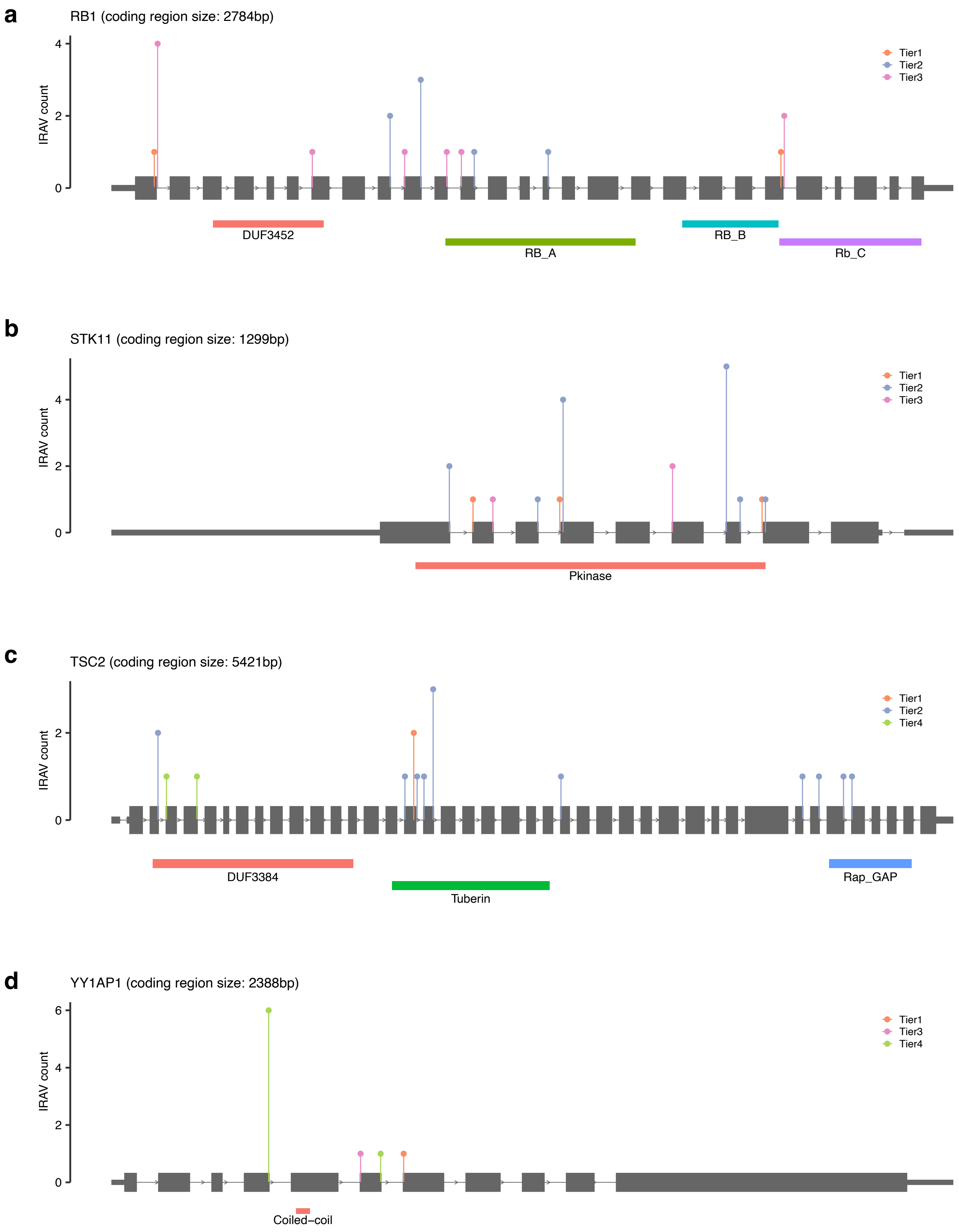

Supplementary Figure 14: Distribution of IRAVs in genes with frequent IRAVs. (a-d) Frequencies of putative pathogenic IRAVs for each rank at each splice-site are shown in (a) *RB1*, (b) *STK11*, (c) *TSC2, and* (d) *YY1AP1*.

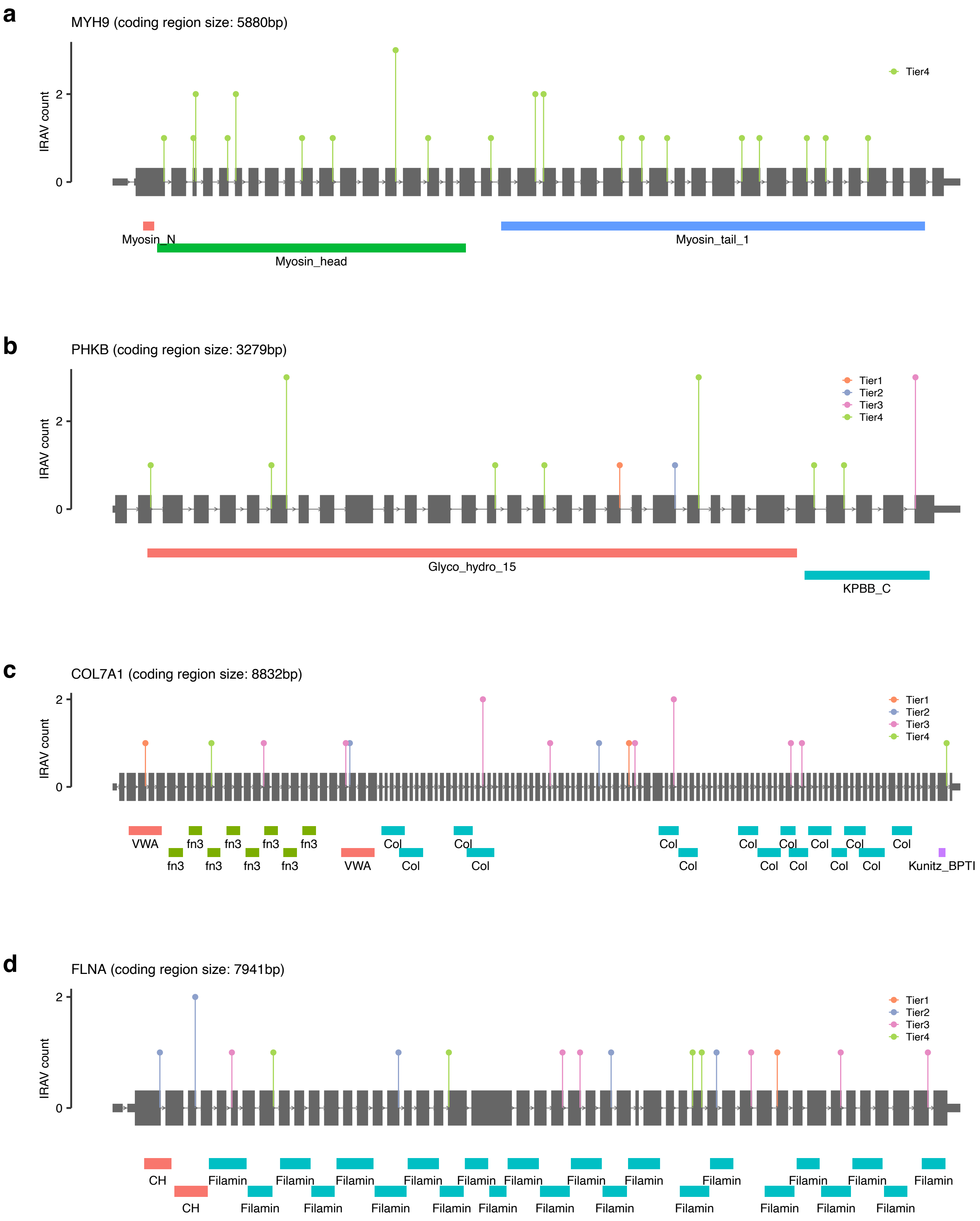

Supplementary Figure 15: Distribution of IRAVs in genes with frequent IRAVs. (a-d) Frequencies of putative pathogenic IRAVs for each rank at each splice-site are shown in (a) *MYH9*, (b) *PHKB*, (c) *COL7A1*, and (d) *FLNA*.

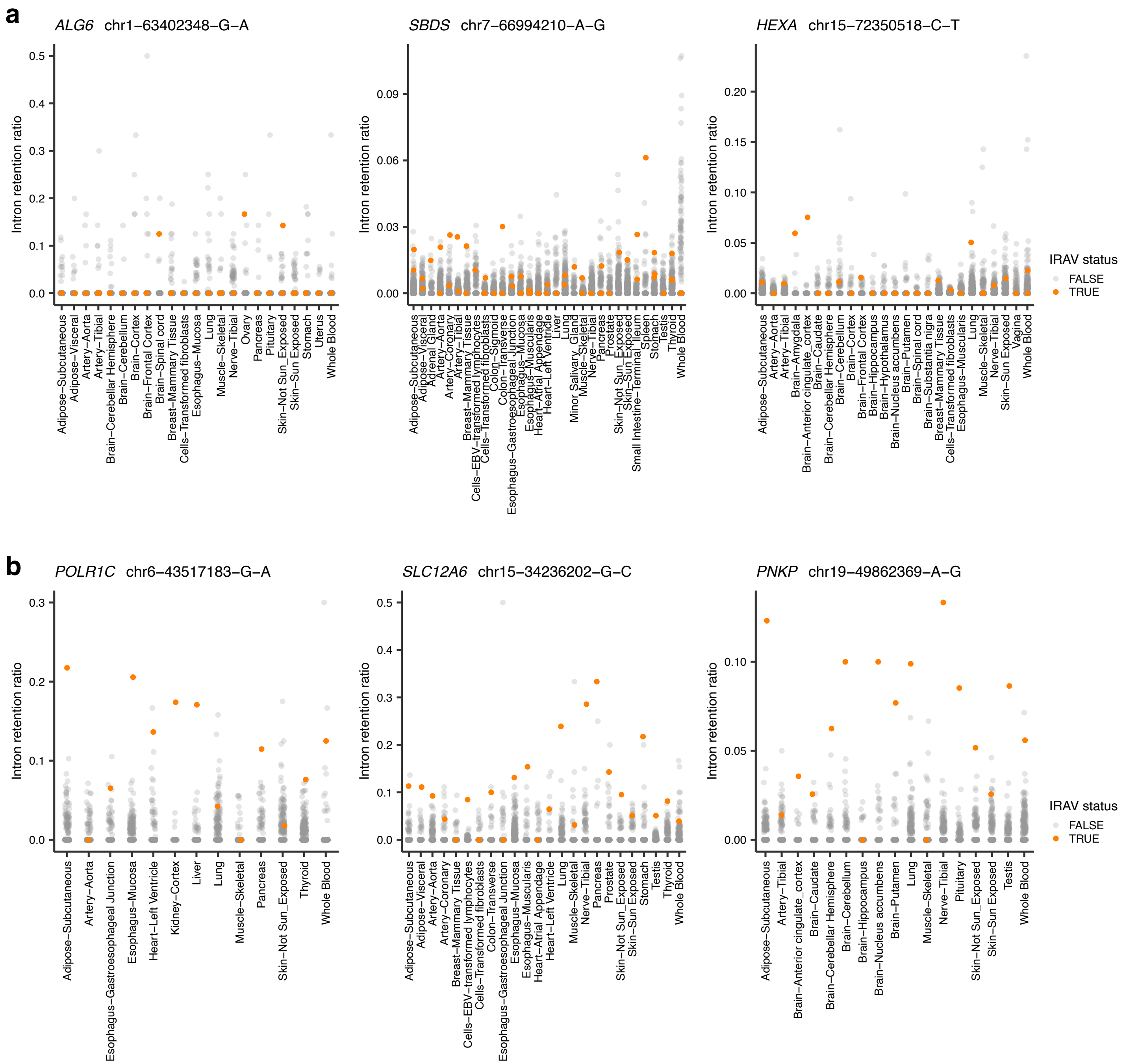

Supplementary Figure 16: (a, b) Relative ratios of corresponding intron retention for samples with and without the IRAVs of Tier1 (a), Tier2 (b) across tissues measured using GTEx transcriptome data. Each point shows an individual colored by IRAV mutation status. The p-values measuring the differences of the intron retention ratios between samples with and without IRAVs via the one-sided Wilcoxon rank sum test for each tissue and integration by Fisher's method were (a) 3.74×10^{-4}, 8.70×10^{-9}, and 1.47×10^{-9}, respectively from the left panel to the right, and (b) 1.51×10^{-16}, 6.09×10^{-49}, and 1.10×10^{-17}, respectively. See also Figure 6a and Supplementary Figure 17.

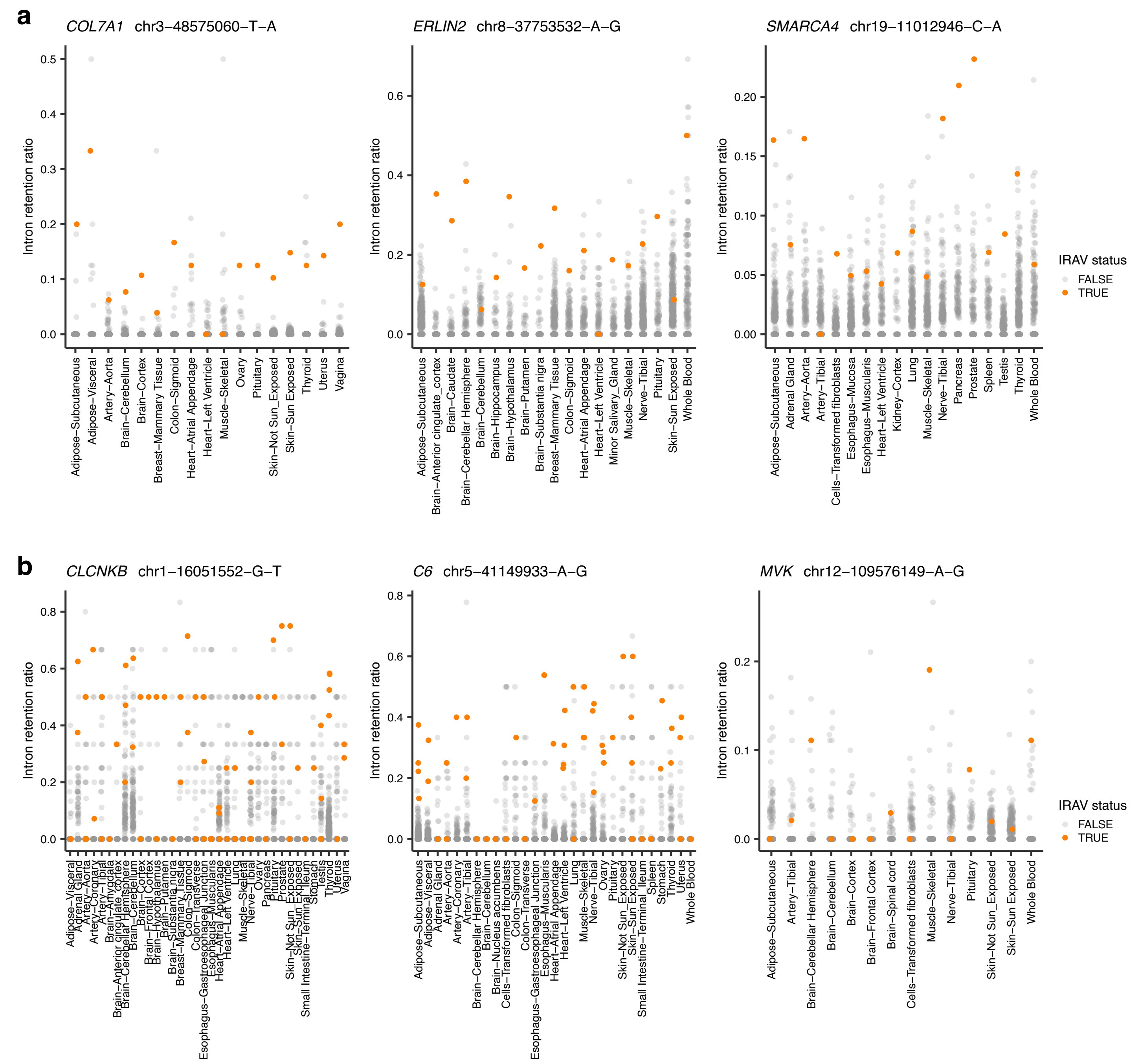

Supplementary Figure 17: (a, b) Relative ratios of corresponding intron retention for samples with and without the IRAVs of Tier3 (a), Tier4 (b) across tissues measured using GTEx transcriptome data. Each point shows an individual colored by IRAV mutation status. The p-values measuring the differences of the intron retention ratios between samples with and without IRAVs via the one-sided Wilcoxon rank sum test for each tissue and integration by Fisher's method were (a) 5.38×10^{-74}, 1.51×10^{-13}, and 2.16×10^{-10}, respectively from the left panel to the right, and (b) 1.43×10^{-41}, 3.22×10^{-67}, and 6.15×10^{-16}, respectively. See also Figure 6a and Supplementary Figure 16.
