# Supplementary material for: Systematic identification of intron retention associated variants from massive publicly available transcriptome sequencing data
Source: Nat Commun. 2022 Sep 29;13:5357. doi: 10.1038/s41467-022-32887-9 (PMC9522810; doi:10.1038/s41467-022-32887-9)
Supplement: Supplementary file 3 — Description of Additional Supplementary Files [file 41467_2022_32887_MOESM3_ESM.pdf]

Description of Additional Supplementary Files

File Name: Supplementary Data 1
Description: List of TCGA samples used in this study and the frequencies of their IRAVs.

File Name: Supplementary Data 2
Description: List of IRAVs affecting cancer-related genes (those registered in Cancer Gene Census) identified in TCGA samples.

File Name: Supplementary Data 3
Description: List of putative pathogenic IRAVs.

File Name: Supplementary Data 4
Description: List of IRAVs related to drug response.
