# Supplementary material for: Systematic identification of intron retention associated variants from massive publicly available transcriptome sequencing data
Source: Nat Commun. 2022 Sep 29;13:5357. doi: 10.1038/s41467-022-32887-9 (PMC9522810; doi:10.1038/s41467-022-32887-9)
Supplement: Supplementary file 8 — Reporting Summary [file 41467_2022_32887_MOESM8_ESM.pdf]

nature portfolio

Corresponding author(s): Yuichi Shiraishi

Last updated by author(s): Aug 9, 2022

Reporting Summary

Nature Portfolio wishes to improve the reproducibility of the work that we publish. This form provides structure for consistency and transparency in reporting. For further information on Nature Portfolio policies, see our Editorial Policies and the Editorial Policy Checklist.

Statistics

For all statistical analyses, confirm that the following items are present in the figure legend, table legend, main text, or Methods section.

n/a	Confirmed	
☐	☒	The exact sample size (*n*) for each experimental group/condition, given as a discrete number and unit of measurement
☒	☐	A statement on whether measurements were taken from distinct samples or whether the same sample was measured repeatedly
☒	☐	The statistical test(s) used AND whether they are one- or two-sided<br>*Only common tests should be described solely by name; describe more complex techniques in the Methods section.*
☒	☐	A description of all covariates tested
☒	☐	A description of any assumptions or corrections, such as tests of normality and adjustment for multiple comparisons
☐	☒	A full description of the statistical parameters including central tendency (e.g. means) or other basic estimates (e.g. regression coefficient) AND variation (e.g. standard deviation) or associated estimates of uncertainty (e.g. confidence intervals)
☒	☐	For null hypothesis testing, the test statistic (e.g. *F*, *t*, *r*) with confidence intervals, effect sizes, degrees of freedom and *P* value noted<br>*Give P values as exact values whenever suitable.*
☒	☐	For Bayesian analysis, information on the choice of priors and Markov chain Monte Carlo settings
☒	☐	For hierarchical and complex designs, identification of the appropriate level for tests and full reporting of outcomes
☒	☐	Estimates of effect sizes (e.g. Cohen's *d*, Pearson's *r*), indicating how they were calculated

Our web collection on statistics for biologists contains articles on many of the points above.

Software and code

Policy information about availability of computer code

Data collection	For Sequence Read Archive (SRA) data, we queried the SRA website (https://www.ncbi.nlm.nih.gov/sra) with the following search term: "platform illumina"[Properties] AND "strategy rna seq"[Properties] AND "human"[Organism] AND "cluster public"[Properties] AND "biomol rna"[Properties]. Then, we extracted samples whose base number is ≥ 1 billion bases, to secure sufficient sequence coverage for reliably detect mutations. We used SRA Toolkit version 2.10.0. First, we performed prefetch command with the "----max-size 100000000" option to download SRA format file. Then, we executed fasterq-dump command with the option "-v --split-files." There were a number of run data that could not be downloaded even after repeated trials probably due to some technical issue. Some of the downloaded sequence data had severe problems such as inconsistencies between two paired-end files, different lengths between sequence letters and base qualities, and so on). In addition, we removed run data whose number of IRAVs was extremely high due to potential DNA contamination and so on.<br><br>For The Cancer Genome Atlas (TCGA) transcriptome data, we used all the available sequencing data at the Genomic Data Commons.
Data analysis	The software used in this paper are listed as below:<br><br>Data download and alignment:<br>SRA Toolkit (version 2.10.0), STAR (version 2.7.2b)<br><br>Intron retention associated variant detection and quantification of intron retention:<br>iravnet (version 0.1.0b15), samtools (version 1.9), bcftools (version 1.9), bedtools (version 2.27.0), Complete-Striped-Smith-Waterman-Library (version 1.1), annot_utils (version 0.3.1), intron_retention_utils (version 0.6.3), MaxEntScan perl wrapper (as of April 21, 2004)<br><br>Performance evaluation of IRAVNet:<br>IRFinder (version 1.3.1), MAJIQ (version 2.3), MMSplice (version 2.2.0), SpliceAIv1.3 variant annotation (as of October 4, 2019).<br><br>Annotation of intron retention associated variants:

ClinVar (as of April 4, 2021), Cancer Gene Census (as of April 9, 2021), ACMG SF list (version 3.0), ClinGen Gene Curation Results (as of April 27, 2021)

For manuscripts utilizing custom algorithms or software that are central to the research but not yet described in published literature, software must be made available to editors and reviewers. We strongly encourage code deposition in a community repository (e.g. GitHub). See the Nature Portfolio guidelines for submitting code & software for further information.

Data

Policy information about availability of data

All manuscripts must include a data availability statement. This statement should provide the following information, where applicable:

- Accession codes, unique identifiers, or web links for publicly available datasets
- A description of any restrictions on data availability
- For clinical datasets or third party data, please ensure that the statement adheres to our policy

The list of intron retention associated variants identified in this study is available through the portal site, IRAVDB (https://iravdb.io/).

Field-specific reporting

Please select the one below that is the best fit for your research. If you are not sure, read the appropriate sections before making your selection.

☒ Life sciences ☐ Behavioural & social sciences ☐ Ecological, evolutionary & environmental sciences

For a reference copy of the document with all sections, see nature.com/documents/nr-reporting-summary-flat.pdf

Life sciences study design

All studies must disclose on these points even when the disclosure is negative.

Sample size	No sample-size calculation was performed. We used as much as we could use from Sequence Read Archive and The Cancer Genome Atlas.
Data exclusions	We excluded some transcriptome sequencing data from Sequence Read Archive due to quality problems (such as inconsistencies between two paired-end files, different lengths between sequence letters and base qualities, and so on).
Replication	Our analysis workflow for detecting intron retention associated variants was executed using Docker containers. Therefore, the analytical result should be reproducible. For mini-gene assay, the experimental results were confirmed by three independent biological replicates and representative results were shown.
Randomization	Since this study had not been planned to identify differences between the allocated groups, randomization is irrelevant.
Blinding	Since this study had not been planned to identify differences between the allocated groups, blinding is irrelevant.

Reporting for specific materials, systems and methods

We require information from authors about some types of materials, experimental systems and methods used in many studies. Here, indicate whether each material, system or method listed is relevant to your study. If you are not sure if a list item applies to your research, read the appropriate section before selecting a response.

Materials & experimental systems

n/a	Involved in the study	
☒	☐	Antibodies
☐	☒	Eukaryotic cell lines
☒	☐	Palaeontology and archaeology
☒	☐	Animals and other organisms
☒	☐	Human research participants
☒	☐	Clinical data
☒	☐	Dual use research of concern

Methods

n/a	Involved in the study	
☒	☐	ChIP-seq
☒	☐	Flow cytometry
☒	☐	MRI-based neuroimaging

Eukaryotic cell lines

Policy information about cell lines

Cell line source(s)	HEK293T cells were obtained from the American Type Culture Collection (ATCC).
Authentication	An aliquot of HEK293T cell lines were authenticated using ATCC DNA fingerprinting.

Mycoplasma contamination	All cell lines are frequently tested for mycoplasma contamination. Cell lines used in this study were verified to be mycoplasma negative before undertaking any experiments with them.
Commonly misidentified lines (See ICLAC register)	No commonly misidentified cell lines were used.
